# The Italian version of the Reflective Functioning Questionnaire: Validity within a sample of adolescents and associations with psychological problems and alexithymia

**DOI:** 10.1002/jclp.23218

**Published:** 2021-07-17

**Authors:** Fabiola Bizzi, Anna Riva, Jessica L. Borelli, Simone Charpentier‐Mora, Monica Bomba, Donatella Cavanna, Renata Nacinovich

**Affiliations:** ^1^ Department of Educational Science University of Genoa Genoa Italy; ^2^ Clinic of Child and Adolescent Neuropsychiatry San Gerardo Hospital‐Monza University of Milan‐Bicocca Monza Italy; ^3^ Department of Psychology and Social Behavior University of California Irvine California USA

**Keywords:** adolescents, mentalizing, psychological problems, psychometric properties, Reflective Functioning Questionnaire

## Abstract

**Objectives:**

This study aims to test the psychometric proprieties of the Reflective Functioning Questionnaire (RFQ) applied to younger (13–16 years) and older (17–20 years) Italian adolescents examining (1) the factorial structure of RFQ; (2) its invariance across age and sex; (3) correlations between RFQ subscale scores, as well as the associations of the RFQ with (4) psychological problems and alexithymia dimensions.

**Methods:**

A cross‐sectional study was conducted with 593 adolescents between the ages of 13 and 20 years old recruited from the community within Italy. These participants completed the RFQ, Symptom Checklist‑90, and Toronto Alexithymia Scale.

**Results:**

The two‐factor structure of the RFQ was confirmed. However, higher internal consistency of RFQ was obtained by removing two items that seemed problematic within this sample. Using a six‐item version of RFQ, the two‐factor structure was invariant across adolescent age and sex. Significant correlations among RFQ subscale scores, and between RFQ subscales with both psychological problems and alexithymia dimensions were found.

**Conclusions:**

Preliminary results reveal a short version of RFQ (six‐item) is a suitable measure to assess mentalizing in adolescents in the Italian context.

## 1 INTRODUCTION

Reflective functioning (RF) is the operationalization of the mental processes underlying the capacity to mentalize (Fonagy et al., 1998), namely the ability to understand and interpret—implicitly and explicitly—one's own and others' behavior as an expression of mental states such as feelings, thoughts, fantasies, beliefs, and desires (Fonagy et al., 2002). Theoretically, RF emerges from the convergence of two theories: first, the development of psychic reality theory (Fonagy & Target, 1996), whereby children progressively move from experiencing inner and outer reality as either equivalent or dissociated toward a more integrated and reflective mode; second, social biofeedback theory (Gergely & Watson, 1996), whereby primary attachment relationships constitute the starting point for emotional self‐awareness and the development of self‐control in infancy. From this perspective, the quality of caregiving a child receives plays an important role: secure attachment context relationships favor the development of RF, while disruptions in attachment relationships, most likely in interaction with environmental and genetic vulnerability, are associated with impairments in mentalizing (Fonagy & Bateman, 2007).

Accordingly, narrative approaches about important attachment relationships have been developed to evaluate RF. The Reflective Functioning Scale (RFS; Fonagy et al., 1998) is the standard instrument designed for that purpose. It is a manual providing rating categories designed to be applied to the interviewee's transcript from the Adult Attachment Interview (George et al., 1996) or the Parent Development Interview (Aber et al., 1985). However, because this assessment tool is time‐ and labor‐intensive, requires highly trained raters, necessarily restricting sample sizes, the development of the Reflective Functioning Questionnaire (RFQ; Fonagy et al., 2016), a self‐report questionnaire measure, has become a suitable tool to assess mentalizing capacity in large samples of adults. This instrument permits the identification of two dimensions of mentalizing assessing Certainty (RFQc) and Uncertainty (RFQu) about the mental states of self and others, reflecting two impairments in RF that are common in many mental disorders such as borderline personality disorder, eating disorder, and depression (i.e., hypermentalizing and hypomentalizing, respectively). Hypermentalizing refers to the tendency to develop excessively detailed models of the mind of oneself that go far beyond the available evidence (Fonagy et al., 2016). Hypomentalizing, by contrast, reflects concrete thinking characterized by an absence or unwillingness to develop nuanced and more complex models of the mind of others and/or the self (Fonagy et al., 2016).

Recently, interest in studying RF in youth has also increased. In this regard, Target et al. (2001) developed the Child Reflective Functioning Scale (CRFS), modeled from the RFS (Fonagy et al., 1998), using transcripts from a semistructured interview, the Child Attachment Interview (Shmueli‐Goetz et al., 2008). Alongside this, an adolescent version of the 46‐item adult RFQ has been adapted for use with adolescents (Reflective Function Questionnaire for Youths [RFQY]; Sharp et al., 2009), while Badoud et al. (2015) have adapted the RFQ (Fonagy et al., 2016) for French adolescents, showing configural invariance of the original two‐factor structure of the RFQ across French‐speaking adolescents and adults, satisfactory reliability and construct validity of the two subscales. This version of the RFQ has also been used with Polish adolescents (Gambin et al., 2021), to test the link between mentalizing difficulties, schizotypal personality features, and thought problems in a sample of community adolescents (Salaminios et al., 2020), as well to investigate the longitudinal associations between RF and empathy on potential changes in externalizing behaviors over time (Morosan et al., 2020). Among Italian samples, the RFQ has not yet been used with adolescents but only validated for adults (Morandotti et al., 2018). This represents a gap in the literature.

### 1.1 RF, adolescence, and psychological adjustment

Adolescence is a sensitive period characterized by rapid changes in biological, psychological, and motivational systems and interpersonal relationships. There is a social reorienting wherein the opinions of peers become more important than those of family members. The psychological adjustment of adolescents is influenced by the interaction between the development of socio‐cognitive capacities and challenges from the environment (Blakemore & Mills, 2014), and mentalizing is one such socio‐cognitive capacity that has gained much attention in the last two decades (Fonagy et al., 2002). Mentalizing develops across adolescence before stabilizing in the early 20s and reflects changes in neurocognitive strategy and/or neuroanatomy (Blakemore & Mills, 2014). This is supported by some studies that find lower mean overall RF scores in adolescents as compared with adult samples (Borelli et al., 2015, 2019; Taubner et al., 2013), as well as studies demonstrating that adolescents' RF is related to their sex (Protic et al., 2020), or otherwise is related to age (Cropp et al., 2019).

Studies on adolescents' RF also suggest that the increase of psychopathology in this transitional period (Kessler et al., 2005) may be linked to mentalization deficits (e.g., Fonagy et al., 2002). Specifically, mentalizing difficulties in adolescence are associated with borderline personality pathology (Bo & Kongerslev, 2017; Duval et al., 2018; Sharp et al., 2013), as well as externalizing (Fonagy & Luyten, 2018; Sharp & Venta, 2012), and internalizing disorders (Bizzi et al., 2019; Chow et al., 2017). Psychopathic traits of adolescents are correlated with aggressive behavior/externalizing symptoms in the case of low or moderate RF (Cropp et al., 2019; Taubner & Curth, 2013). In addition, several studies (e.g., Badoud et al., 2015; Gambin et al., 2021; Morosan et al., 2020; Salaminios et al., 2020) using the RFQ version for adolescents show that higher scores on the Uncertainty subscale (i.e., uncertainty about mental states of self and others) and lower scores on the Certainty subscale (i.e., certainty about mental states of self and others) are associated with higher difficulties in emotion regulation, higher levels of alexithymia, borderline personality traits, schizotypal features, as well as internalizing and externalizing symptoms in adolescence.

Although these previous studies have highlighted a link between mentalizing deficits and clinical problems and have also examined the distinctions between mentalizing in adolescence as compared with adulthood, it remains to be seen whether mentalizing varies by according to age and sex within adolescence and whether difficulties in mentalizing may constitute a vulnerability factor also in healthy adolescents. If the RFQ is related to age, sex, clinical variables, and alexithymia within a community sample in Italy, it would establish that is an important construct to study within this population. Similarly, the age and sex differences would help to understand how RF is distributed within this culture and help to know potential confounds that should be measured in future studies. Therefore, this study aims to fill these gaps by testing the psychometric properties of RFQ in early and older adolescents.

### 1.2 Aims and hypotheses

Since the RFQ was developed in the English language (Fonagy et al., 2016), and has since been translated into French and administered to adolescents in France (Badoud et al., 2015) and in some European countries (Gambin et al., 2021; Morosan et al., 2020; Salaminios et al., 2020), our first goal was to administer the RFQ within Italy, and to test preliminary hypotheses regarding its psychometric properties among two groups of adolescents, younger (13–16 years) and older (17–20 years) adolescents. Specifically, we expected to find evidence for a two‐factor structure of the RFQ, referring respectively to the degree of subjective confidence (i.e., Certainty, RFQc) or doubt (i.e., Uncertainty, RFQu) that actions are mentally driven. Furthermore, we predicted that this two‐factor structure would be invariant across (i) younger and older adolescents, (ii) male and female adolescents. Subsequently, following prior validity work on the French version of RFQ (Badoud et al., 2015), we sought to examine correlations between RFQ subscale scores with clinical variables (i.e., psychological problems), as well as psychological capacities (i.e., alexithymia dimensions) that have been previously linked with RF both theoretically and empirically. We hypothesized that there would be negative associations between the degree of certainty concerning mental states (RFQc), psychological problems, and alexithymia dimensions, as well as positive associations between these variables and the degree of uncertainty concerning mental states (RFQu).

## 2 MATERIALS AND METHODS

### 2.1 Participants

We recruited *N* = 593 Italian adolescents (334 [56%] males) between the ages of 13 and 20 (*M* = 16.36, SD = 1.57) from the community. All participants were of national origin and had heterosexual parents. Inclusion criteria included being an adolescent within our target age range (between 13 and 20 years) and speaking fluent Italian. The final sample was divided by age into a group of 378 younger adolescents (209 males, aged 13–16 years old; *M*_AGE_ = 15.35, SD_AGE_ = 0.87) and a group of 215 older adolescents (125 males, aged 17–20 years old; *M*_AGE_ = 18.12, SD_AGE_ = 0.73). Younger and older adolescents differed significantly in terms of participant age (*t*(592) = −39.53, *p* = .000), but they did not differ significantly in terms of participant sex (male: 55% in younger vs. 58% in older adolescents; *χ* ^2^ = 0.45, *p* = .501) or family composition (biparental family: 87% in younger vs. 90% in older adolescents; monoparental family: 13% in younger vs. 10% in older adolescents; *χ* ^2^ = 1.15, *p* = .283).

### 2.2 Measures

The *Reflective Functioning Questionnaire* (RFQ; Fonagy et al., 2016) is a questionnaire used to evaluate mentalizing abilities by measuring the degree of certainty and uncertainty with which individuals utilize mental state information to understand their own and others' behavior. The Certainty about Mental States (RFQc) subscale consists of six items focusing on the extent to which individuals disagree with statements such as “I don't always know why I do what I do.” All items are scored by participants on a 7‐point Likert‐type scale, ranging from “completely disagree” to “completely agree.” Items are subsequently rescored to capture more extreme levels of certainty, so that very low agreements on this scale reflect hypermentalizing, while some agreement reflects adaptive levels of certainty about mental states. To this end, these items are recorded to 3, 2, 1, 0, 0, 0, 0. The Uncertainty about mental states (RFQu) subscale, which in the extreme captures hypomentalizing, also consists of six items scored on the same 7‐point Likert‐type scale. Responses to items such as “Sometimes I do things without really knowing why,” are recoded to 0, 0, 0, 0, 1, 2, 3, again to ensure that high scores reflected a stance characterized by an almost complete lack of knowledge about mental states, while lower scores reflect an acknowledgment of the opaqueness of one's mental states and that of others, typical of genuine mentalizing. The items are described in Table 1. For this study, we used the Italian version of the test provided by Fonagy et al. (2016) and available at https://www.ucl.ac.uk/psychoanalysis/research/reflective-functioning-questionnaire-rfq.

**Table 1:** Items and factors of the Reflective Functioning Questionnaire

Item	Factor c	Factor u
1. People's thoughts are a mystery to me (original item 1)	RFQc1	
2. I don't always know why I do what I do (original item 17)	RFQc2	RFQu2
3. When I get angry I say things without really knowing why I am saying them (original item 22)	RFQc3	
4. When I get angry I say things that I later regret (original item 29)	RFQc4	RFQu4
5. If I feel insecure I can behave in ways that put others' backs up (original item 35)	RFQc5	RFQu5
6. Sometimes I do things without really knowing why (original item 36)	RFQc6	RFQu6
7. I always know what I feel (original item 8)		RFQu7
8. Strong feelings often cloud my thinking (original item 27)		RFQu8

The *Symptom Checklist‑90–Revised* (SCL‑90‑R; Derogatis & Savitz, 1999; Prunas et al., 2012) is a self‐report questionnaire designed to assess psychological problems and psychopathological symptoms in individuals 13 years and older. This original measure, as well as the validated Italian version (Prunas et al., 2012) we used, consist of 90 items rated on a 5‐point Likert scale that assess nine symptom dimensions (somatization [SOM], obsessive‐compulsive [OC], interpersonal sensitivity [INT], depression [DEPR], anxiety [ANX], hostility [HOS], phobic anxiety [PHOB], paranoid ideation [PAR], psychoticism [PSY]). In this study, the internal consistency has been reported as good for all subscales (Cronbach's alpha values between .70 and .94).

The *Toronto Alexithymia Scale* (TAS‐20; Bagby et al., 1994; Bressi et al., 1996) is a 5‐point Likert‐type self‐report 20‐item questionnaire to assess the alexithymia (i.e., a personality trait characterized by the subclinical inability to identify and describe emotions experienced by one's self or others) in the total level and the three aforementioned factors, as difficulties in identifying feelings (DIF), difficulties in describing feelings (DDF), and lack of focus on internal emotional experiences (EOT). In this study, the internal consistency has been reported as good for all subscales (Cronbach's alpha values between .81 and .89).

### 2.3 Procedure

The procedure was approved by the local University Research Ethical Committee and complied with the ethical standards of the international scientific community. Data were collected within 2020. The adolescents were recruited from two high schools (a high school and a technical institute) in northern Italy (from the first to the fifth grade); every student in each grade was contacted at school by a medical resident belonging to the research team for a total of 605 participants, of which only 12 did not participate because they were absent at the time of the data collection. Therefore, the final sample (*N* = 593) represented 98% of those eligible.

All the parents and participants were informed of the research goals. In line with ethical requirements, it was emphasized that participants' cooperation was voluntary and that their answers were confidential and used only for the study. Written informed consent was received from the participants (and the parents of adolescents if they were younger than 18 years old). The assessment was administered to the adolescents at school in a data collection session lasting around 45 min.

### 2.4 Statistical analyses

Statistical analyses were carried out with Statistical Package for the Social Sciences (SPSS, Version 24.0; IBM Corp.) and JASP (JASP Team, 2020). Demographic variables were assessed using descriptive statistics (i.e., *χ* ^2^ test and Student's *t* tests). According to our aims, analyses were assessed following three steps. First, we examined the supposed two‐factorial structure of RFQ using confirmatory factor analysis (CFA). Second, we used multigroup CFA to investigate the factorial invariance of RFQ in both groups (male vs. female adolescents; younger vs. older adolescents) testing for configural, metric, scalar, and strict measurement invariance. We used fit indices to test model fit using cut‐off values generally indicating a good fit (Hu & Bentler, 1999): *χ* ^2^/*df* ratio (<3 acceptable), comparative fit index (CFI over 0.90 acceptable), Tucker–Lewis fit index (TLI over 0.90 acceptable), root mean square error of approximation (RMSEA between 0.05 and 0.08 acceptable), and standardized root mean squared residual (SRMR < 0.08 acceptable). A comparison between different factor solutions has been made with a *χ* ^2^ difference test and a drop in CFI greater than 0.005 (Chen, 2007). Third, Pearson correlations were used to test the association between RFQ, demographic features, clinical variables (psychological problems), and alexithymia dimensions.

## 3 RESULTS

### 3.1 Confirming internal structure and measurement invariance of CRFS

Initially, we explored items characteristics of RFQ (see Table 2), which demonstrated the items' adequate distributional characteristics. Then, we examined the two‐factorial structure of RFQ proposed by Fonagy et al. (2016) with CFA. To do so, we first tested the two‐factor model which did not yield a satisfactory factor solution (Model 1): *χ* ^2^ = 632.18, *χ* ^2^/*df* = 11.93, CFI = 0.68, TLI = 0.60, RMSEA = 0.14, SRMR = 0.09. However, Model 1 significantly improved by allowing error correlation between several items with similar wording (c2–u2, c3–u8, c4–u4, c5–u5, c6–u6) and modification indices values higher above 10 (Byrne, 2016) resulting in a good fit model (Model 1a): *χ* ^2^ = 172.10, *χ* ^2^/*df* = 3.58, CFI = 0.93, TLI = 0.90, RMSEA = 0.07, SRMR = 0.06. All the standardized coefficients for this two‐factor model were statistically significant except for one item (see Table 3).

**Table 2:** Item and score characteristics of the Reflective Functioning Questionnaire

Item	*N*	*M*	SD	Range	Skew	Kurtosis
RFQc1	593	0.86	1.03	0–3	0.81	−0.68
RFQc2	593	0.56	0.83	0–3	1.30	0.70
RFQc3	593	1.03	1.12	0–3	0.52	−1.22
RFQc4	593	0.91	1.12	0–3	0.71	−1.05
RFQc5	593	0.72	1.06	0–3	1.11	−0.30
RFQc6	593	0.67	1.05	0–3	1.25	0.04
RFQu2	593	0.78	1.01	0–3	0.93	−0.51
RFQu4	593	0.79	1.08	0–3	1.01	−0.45
RFQu5	593	0.89	1.08	0–3	0.80	−0.79
RFQu6	593	0.99	1.13	0–3	0.69	−1.01
RFQu7	592	1.34	1.24	0–3	0.16	−1.60
RFQu8	593	0.50	0.90	0–3	1.64	1.43
RFQc	593	0.79	0.57	0–2.5	0.78	0.01
RFQu	593	0.88	0.52	0–2.83	0.71	0.19

**Table 3:** Factor loadings and factors covariance of original two‐factor model of the Reflective Functioning Questionnaire

Factor	Indicator	Estimate	SE	*Z*	*p*	Standard estimate
RFQ_c	RFQc1	0.25	0.05	5.07	<.001	0.24
	RFQc2	−0.09	0.04	−2.44	.014	−0.11
	RFQc3	0.54	0.05	10.69	<.001	0.48
	RFQc4	0.82	0.05	17.08	<.001	0.73
	RFQc5	0.58	0.04	12.87	<.001	0.55
	RFQc6	0.67	0.04	14.66	<.001	0.64
RFQ_u	RFQu2	0.05	0.04	1.13	.257	0.05
	RFQu4	−0.79	0.05	−17.10	<.001	−0.72
	RFQu5	−0.53	0.05	−11.29	<.001	−0.49
	RFQu6	−0.82	0.05	−17.08	<.001	−0.72
	RFQu8	−0.43	0.04	−10.77	<.001	−0.48
	RFQu7	0.42	0.06	7.34	<.001	0.34
					Factors covariance (SE)	
RFQ_c–RFQ_u						0.78 (0.03)

Internal consistency reliability in the total sample for the two scales was not acceptable: uncertainty scale (*α* = .34, mean inter‐item correlation = 0.09), certainty scale (*α* = .54, mean inter‐item correlation = 0.14). Internal consistency reliability in the two adolescents' groups was also not acceptable: younger adolescents (uncertainty scale, *α* = .38, mean inter‐item correlation = 0.10; certainty scale, *α* = .54, mean inter‐item correlation = 0.14); older adolescents (uncertainty scale, *α* = .28, mean inter‐item correlation = 0.07; certainty scale, *α* = .54, mean inter‐item correlation = 0.14). In addition, internal consistency could be improved by the deletion of some items that seem problematic: item c2 (*α* = .65) in Certainty scale; items u2 (*α* = .40) and item u7 (α = 0.57) in Uncertainty scale.

As the scale's reliability was not supported, we deleted items 2 and 7, testing a six‐item RFQ model, which did not yield an initial satisfactory factor solution (model 2): *χ* ^2^ = 370.31, *χ* ^2^/*df* = 14.24, CFI = 9.77, TLI = 0.68, RMSEA = 0.15, SRMR = 0.08. However, Model 2 significantly improved by allowing error correlation between several items with similar wording (c4–u4, c3–u8, c5–u5, c6–u6) and modification indices values higher above 10 (Byrne, 2016) resulting in a good fit model (Model 2a): *χ* ^2^ = 61.28, *χ* ^2^/*df* = 2.78, CFI = 0.97, TLI = 0.96, RMSEA = 0.05, SRMR = 0.04. All the standardized coefficients for this two‐factor model were statistically significant (see Table 4).

**Table 4:** Factor loadings and factors covariance of six‐item two‐factor model of the Reflective Functioning Questionnaire

Factor	Indicator	Estimate	SE	*Z*	*p*	Standard estimate
RFQ_c	RFQc1	0.24	0.05	4.85	<.001	0.23
	RFQc3	0.52	0.05	10.31	<.001	0.46
	RFQc4	0.84	0.05	17.09	<.001	0.74
	RFQc5	0.56	0.04	12.38	<.001	0.53
	RFQc6	0.66	0.05	14.50	<.001	0.63
RFQ_u	RFQu4	0.81	0.05	17.47	<.001	0.75
	RFQu5	0.50	0.05	10.83	<.001	0.47
	RFQu6	0.82	0.05	16.96	<.001	0.72
	RFQu8	0.42	0.04	10.63	<.001	0.47
					Factors covariance (SE)	
RFQ_c–RFQ_u						−0.75 (0.03)

Finally, we performed a six‐item RFQ and original RFQ model comparison in which six‐item RFQ model showed a better fit than the previous original RFQ model (*χ* ^2^(26) = 110.82, *p* < .001, ΔCFI = 0.04). Internal consistency reliability of six‐item RFQ in the total sample for the two scales was good: uncertainty scale (*α* = .69, mean inter‐item correlation = 0.37), certainty scale (*α* = .65, mean inter‐item correlation = 0.27). The internal consistency of the scale in the two adolescent groups was also good: younger adolescents (uncertainty scale, *α* = .69, mean inter‐item correlation = 0.365; certainty scale, *α* = .64, mean inter‐item correlation = 0.26); older adolescents (uncertainty scale, *α* = .70, mean inter‐item correlation = 0.37; certainty scale, *α* = .66, mean inter‐item correlation = 0.28).

Measurement invariance of Model 2a between younger (*N* = 378) and older (*N* = 215) adolescents and males (*N* = 334) and females (*N* = 259) adolescents were tested with a multi‐group CFA with increasingly restrictive models (see Table 5). About younger and older adolescents, we tested configural invariance (Model 3), metric invariance (Model 4), scalar invariance (Model 5), and strict invariance (Model 6) obtaining a good model fit in every model (see Table 5 for the different models' fit values), and thereby our findings confirm factorial invariance across both younger and older adolescents' groups. About male and female adolescents, we tested configural invariance (Model 7), metric invariance (Model 8), scalar invariance (Model 9), and strict invariance (Model 10), obtaining a good model fit in every model (see Table 5 for the different models' fit values), and thereby our findings confirm factorial invariance across both male and female adolescents' groups. In total, our findings confirm the two‐factorial solution of six‐item RFQ and indicate factorial invariance across both younger and older adolescents' groups and male and female adolescents' groups.

**Table 5:** Fit statistics CFA models

Models	*χ* ^2^	*df*	*p*	*χ* ^2^/*df*	RMSEA	TLI	CFI	SRMR
Model 1	632.18	53	<.001	11.93	0.14	0.60	0.68	0.09
Model 1a	172.10	48	<.001	3.58	0.07	0.90	0.93	0.06
Model 2	370.31	26	<.001	14.24	0.15	0.68	0.77	0.08
Model 2a	61.28	22	<.001	2.78	0.05	0.96	0.97	0.04
Model 3	75.57	44	.002	1.72	0.05	0.96	0.98	0.04
Model 4	79.03	53	.01	1.49	0.04	0.98	0.98	0.05
Model 5	85.41	60	.01	1.42	0.04	0.98	0.98	0.05
Model 6	98.05	73	.02	1.34	0.03	0.98	0.98	0.05
Model 7	90.66	44	<.001	2.06	0.06	0.95	0.97	0.04
Model 8	107.14	53	<.001	2.02	0.06	0.95	0.96	0.05
Model 9	114.59	60	<.001	1.90	0.055	0.96	0.96	0.05
Model 10	128.60	73	<.001	1.76	0.051	0.96	0.96	0.06

Abbreviations: CFA, confirmatory factor analysis; CFI, comparative fit index; RMSEA, root mean square error of approximation; SRMR, standardized root mean square residual; TLI, Tucker–Lewis index.

### 3.2 Correlations with demographic variables, psychological problems, and alexithymia dimensions

Means and standard deviation of SCL‐90 (to assess psychological problems) and TAS‐20 (to investigate alexithymia dimension) are reported in Table 6.

**Table 6:** Means and standard deviation of SCL‐90 and TAS‐20

	*N*	*M*	SD
SCL90 SOM	593	40.66	7.24
SCL90 OC	593	40.22	8.27
SCL90 INT	593	40.53	7.26
SCL90 DEPR	593	40.48	8.07
SCL90 ANX	593	41.26	7.82
SCL90 HOS	593	42.41	7.64
SCL90 PHOB	593	44.09	5.92
SCL90 PAR	592	40.81	7.53
SCL90 PSY	593	41.97	6.25
TAS_DIF	593	15.98	5.52
TAS_DDF	593	14.24	4.25
TAS_EOT	593	20.99	4.58

Abbreviations: ANX, anxiety; DDF, difficulties in describing feelings; DEPR, depression; DIF, difficulties in identifying feelings; EOT, lack of focus on internal emotional experiences; HOS, hostility; INT, interpersonal sensitivity; OC, obsessive‐compulsive symptoms; PAR, paranoid ideation; PHOB, phobic anxiety; PSY, psychoticism; SOM, somatic symptoms; SCL‐90, Symptom Checklist‑90; TAS‐20, Toronto Alexithymia Scale.

Using the six‐item version of RFQ, neither RFQ subscale was related to demographic features (age, sex, family composition). Correlations with psychological problems and alexithymia dimensions obtained from self‐report measures are shown in Table 7. Congruent with expectations, the RFQc subscale of the six‐item version of RFQ was negatively correlated with all clinical variables (i.e., psychological problems) assessed with the SCL‐90, and with alexithymia in all subscales of TAS‐20. The RFQu was positively correlated with all subscales of the SCL‐90 and with alexithymia in all subscales assessed with the TAS‐20.

**Table 7:** Correlations between the RFQ (six‐item version), psychological problems (SCL‐90), and alexithymia dimensions (TAS‐20)

	SCL‐90 SOM	SCL‐90 OC	SCL‐90 INT	SCL‐90 DEPR	SCL‐90 ANX	SCL‐90 HOS	SCL‐90 PHOB	SCL‐90 PAR	SCL‐90 PSY	TAS DIF	TAS DDF	TAS EOT
RFQc	−0.14**	−0.18**	−0.18**	−0.18**	−0.12**	−0.11*	−0.12*	−0.14**	−0.14**	−0.42**	−0.33**	−0.21**
RFQu	0.17**	0.27**	0.27**	0.25**	0.21**	0.24**	0.22**	0.23**	0.17**	0.36**	0.25**	0.13**

Abbreviations: ANX, anxiety; DDF, difficulties in describing feelings; DEPR, depression; DIF, difficulties in identifying feelings; EOT, lack of focus on internal emotional experiences; HOS, hostility; INT, interpersonal sensitivity; OC, obsessive‐compulsive symptoms; PAR, paranoid ideation; PHOB, phobic anxiety; PSY, psychoticism; RFQ, Reflective Functioning Questionnaire; SOM, somatic symptoms; SCL‐90, Symptom Checklist‑90; TAS‐20, Toronto Alexithymia Scale.

* *p* < .05.

** *p* < .01.

## 4 DISCUSSION

The present study examined the psychometric proprieties of the Italian versions of the RFQ with a community sample of adolescents. We first predicted we would find evidence for a two‐factor structure, referring respectively to the degree of subjective confidence (i.e., certainty, RFQc) or doubt (i.e., uncertainty, RFQu). Consistent with our expectations and with prior research (Badoud et al., 2015; Fonagy et al., 2016), results revealed a two‐factor structure (RFQc and RFQu subscales) of the RFQ. Therefore, our study revealed support for these different aspects of mentalizing in Italian adolescents. Further, we predicted that the two‐factor structure of RFQ in adolescence was invariant across younger (13–16 years) and older adolescents (17–20 years), as well as across males and females. The results confirmed the expectations. However, higher internal consistency of RFQ was obtained by removing two items (item 2 and item 7) that seemed problematic within this population. In particular, item 2 was formulated with a negative sentence, and for cultural reasons, this item might be challenging to understand and might not assess what it is supposed to assess. In addition, the use of the adverb “always” in both items could confuse the answers and the inclusion of the word “always” could be more complicated also in the sentence with the negation where “not always” could correspond to “sometimes.” Therefore, from this perspective, a six‐item RFQ model significantly improved the internal structure of RFQ as well as its clarity in understanding. Despite the multiple developmental changes that are inherent to adolescence and the differences in terms of sociodemographic data (i.e., sex) found in previous studies of reflective ability (Cropp et al., 2019; Protic et al., 2020), no differences were found comparing younger and older adolescents nor males and females on the RFQ. This finding, which contradicts the theoretical framework of mentalization may suggest that the RFQ is not sufficiently sensitive to sex differences or may be due to the characteristics of our sample and should be verified in future studies. Overall, our findings show that the six‐item version of RFQ is a suitable measure to assess mentalizing capacity in adolescents on a large scale (i.e., easily applied with large samples).

Our last hypothesis was that the RFQ subscale scores were related to clinical variables (i.e., psychological problems) and psychological capacity (i.e., alexithymia). Results revealed negative associations relatively modest in size (Cohen, 1988) between the degree of certainty concerning mental states (RFQc) with psychological problems and alexithymia dimensions, as well as a reverse pattern of associations between the factors listed above and the degree of uncertainty about mental states (RFQu). These results are consistent with the mentalizing framework (Fonagy et al., 2016) suggesting that the RFQ_u may be a good marker of typical features associated with clinical problems, while the RFQ_c may be a marker of psychological adjustment for adolescents. In this sense, the poorer an adolescent's mentalizing, the more problems concerning affect dysregulation and emotional problems can be expected (Fonagy et al., 2016). The link between RFQ_u and clinical problems is in line with the literature: Morosan et al. (2020) found associations with externalizing behavior and internalizing problems, Badoud et al. (2015) with borderline traits, and Salaminios et al. (2020) with interpersonal schizotypal manifestations although using other clinical measures respect to SCL‐90. Besides, in contrast to Badoud et al. (2015) findings, we found associations between all subscales of the alexithymia questionnaire (also the externally oriented thoughts subscale) and each of the two RFQ subscales, suggesting that as discovered by Gambin et al. (2021), despite using the Difficulties in Emotion Regulation Scale rather than the TAS‐20, mentalizing abilities play a very important role in emotion regulation by providing awareness and understanding of own and others mental states, as well as ability to correctly identify and manage emotional states. Similarly, but in the opposite direction, adolescents' poor mentalizing seems to reflect general emotional distress, including a difficulty in recognizing and distinguishing between feelings and bodily sensations, in describing feelings to others, and restricted imagination, marked by the paucity of fantasies, dreams, and daydreaming. However, these findings need to be interpreted with caution as the clinical problems and alexithymia do not reach the clinical cut‐off.

We believe it is useful to contextualize the contribution of our study in terms of its strengths and limitations. A strength of this study is the large number of participants, as well as the focus on different age ranges within the broader adolescent phase. However, some methodological limitations must be mentioned. First, our analyses and conclusions are exclusively based on self‐report questionnaires. As a result, studies are now needed to investigate mentalizing in adolescence using multi‐method approaches. A further weakness is the lack of an appropriate criterion in comparing our results; therefore, our conclusion that the RFQ measures RF can be only provisional (Badoud et al., 2018). Besides, the results on the six‐item version of RFQ need to be replicated within clinical samples, testing if this version is a valid method to assess mentalizing also in clinical adolescents. In this regard, future studies may explore the pattern highlighted in this study between mentalization, alexithymia, and psychological problems to illustrate the absence or presence or greater strength of these relationships in clinical populations. Moreover, this study does not include scales measuring externalizing constructs and other personality disorder traits (i.e., borderline or schizotypal features) that previous literature has shown to be linked to mentalizing difficulties. Lastly, RFQ's overlapping of the questions which make up the two scales might be an issue, but nonetheless, our findings suggest that RFQ subscales are associated with psychological problems and alexithymia dimensions in opposite directions, suggesting a negative covariance in accordance with prior studies (Badoud et al., 2015; Fonagy et al., 2016; Morandotti et al., 2018). These limitations help to point the way toward the next frontier in this line of research.

In sum, our results provide preliminary evidence that in the Italian context a short version of RFQ (six‐item RFQ) holds solid psychometric properties, and it can be used to assess mentalizing in adolescence. Besides, this study provides basic information about the assessment RF tool in healthy adolescents supporting that the six‐item RFQ version can evaluate mentalizing independently to age and sex within adolescence and that is able to individuate mentalizing difficulties which may contribute (or limit) the vulnerability to psychological problems and alexithymia through exploring of specific RFQ subscales. In other words, this instrument can be considered as a screening tool able to measure individuals' reflective abilities in adolescence, detecting possible difficulties or skills in healthy samples. In this way, this measure represents a very useful tool in terms of providing information on reflective abilities in adolescence and has crucial utility in the assessment process. Mentalizing abilities could be an important target for interventions aimed at improving emotion regulation strategies and psychological well‐being in youth (Gambin et al., 2021). We suggest a systematic use of the RFQ (opportunely adapted to the different contexts) to increase our knowledge about mentalizing in adolescence.

## CONFLICT OF INTERESTS

The authors declare that there are no conflict of interests.

## ETHICS STATEMENT

All procedures were in accordance with the ethical standards of the institutional research committee, the international standards, and the 1964 Helsinki Declaration and its later amendments. Informed consent was obtained from all individual participants included in the study (and the parents of adolescents if below 18 years old).

### PEER REVIEW

The peer review history for this article is available at https://publons.com/publon/10.1002/jclp.23218

## Data Availability

The data that support the findings of this study are available on request from the corresponding author.
